# Effect of cyclodextrin complexation on phenylpropanoids’ solubility and antioxidant activity

**DOI:** 10.3762/bjoc.10.241

**Published:** 2014-10-06

**Authors:** Miriana Kfoury, David Landy, Lizette Auezova, Hélène Greige-Gerges, Sophie Fourmentin

**Affiliations:** 1 Bioactive Molecules Research Group, Doctoral School of Science and Technology, Department of Chemistry and Biochemistry, Faculty of Sciences-2, Lebanese University, Lebanon; 2 Univ Lille Nord de France, F-59000 Lille, France; 3 ULCO, UCEIV, F-59140 Dunkerque, France

**Keywords:** antioxidant activity, complexation efficiency, cyclodextrins, formation constant, phenylpropanoids, solubility

## Abstract

The complexation abilities of five cyclodextrins (CDs) with seven phenylpropanoids (PPs) were evaluated by UV–visible spectroscopy, phase solubility studies and molecular modeling. Formation constants (*K*_f_), complexation efficiency (CE), PP:CD molar ratio, increase in formulation bulk and complexation energy were assessed. All complexes exhibited a 1:1 stoichiometry but their stability was influenced by the nature and the position of the phenyl ring substituents. A relationship between the intrinsic solubility of guests (*S*_0_) and the solubilizing potential of CD was proposed. Molecular modeling was used to investigate the complementarities between host and guest. Finally, the antioxidant activity of encapsulated PPs was evaluated by scavenging of the stable DPPH radical.

## Introduction

Phenylpropanoids (PPs), produced through the shikimic acid pathway, are one of the major groups of natural compounds. They could be found in a wide variety of plants (clove, anise, basil, tarragon, fennel, parsley, cinnamon, etc). PPs play a vital role in plants integrity and defense against biotic or abiotic stresses and are considered as “secondary metabolites” [1]. They are divided in several major classes such as coumarins, flavonoids, phenylpropenes and hydroxycinnamic acids [2]. PPs have an important antioxidant activity and therefore are considered to have beneficial effects on human health [3–4]. PPs are also known to have antibacterial, antifungal and anti-inflammatory properties [5–7]. However, they have restricted applications as pharmaceutical products and food preservatives because they have limited water solubility, stability and poor bioavailability [8–9]. Thus, their encapsulation may enhance their apparent solubility without losing their structural integrity and bioactivity.

During the past years, cyclodextrins (CDs) have been widely used as encapsulating agents to enhance the solubility, stability, release and bioavailability of natural compounds [10–13]. CDs are a family of cyclic oligosaccharides obtained from enzymatic degradation of starch. The most common native CDs are α-cyclodextrin (α-CD), β-cyclodextrin (β-CD) and γ-cyclodextrin (γ-CD) composed of six, seven and eight (α-1,4)-linked α-D-glucopyranose units, respectively. Owing to their molecular structure, consisting of a hydrophilic outer surface and a hydrophobic cavity, CDs can form inclusion complexes with organic compounds which enter partly or entirely into their cavity. Inclusion complexes can be obtained either in solution or in solid state [14]. The relative stabilities of inclusion complexes are governed by different factors such as hydrogen bonding, hydrophobic interactions, solvation effects as well as the guest molecule's space filling ability [15–16]. CD derivatives are also of great importance, since they generally have higher aqueous solubility than native CDs and enhanced binding affinities and selectivity [17].

The aim of this study was to investigate the binding ability of five CDs with seven naturally occurring PPs. Formation constants (*K*_f_) of CD/PP inclusion complexes were calculated by UV–visible spectroscopy and phase solubility studies. The solubilizing effects of CDs was investigated by determining their complexation efficiency (CE) [18–19]. A theoretical molecular modeling study has been realized to estimate the complexation energies and illustrate the most favorable inclusion complex structure. Finally, the effect of complexation on the radical scavenging activity of PPs was investigated.

## Results and Discussion

### Formation constants (*K*_f_)

**Figure 1:**
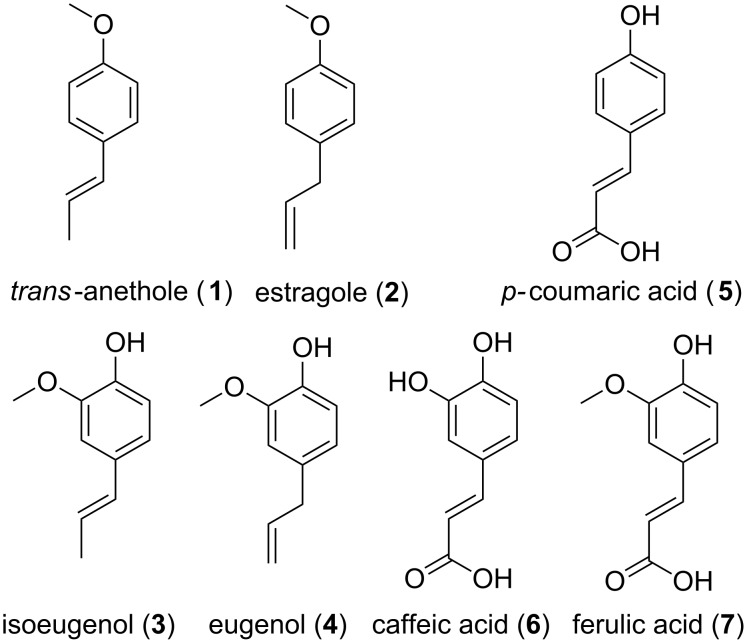
Chemical structure of studied phenylpropanoids (PPs).

The complexation behavior of PPs including four phenylpropenes (*trans-*anethole (**1**), estragole (**2**), isoeugenol (**3**) and eugenol (**4**)) and three hydoxycinnamic acids (*p-*coumaric acid (**5**), caffeic acid (**6**) and ferulic acid (**7**)) (Figure 1) was investigated with α-CD, β-CD, hydroxypropyl-β-cyclodextrin (HP-β-CD), randomly methylated β-cyclodextrin (RAMEB) and a low methylated β-cyclodextrin (CRYSMEB). These PPs were selected mainly according to their structural homology. Moreover, they cover a sufficiently relevant range of solubility and hydrophobicity (Table 1).

Formation constant (*K*_f_) values were calculated using a UV–visible spectral displacement method [20–21]. This method implies first the characterization of the inclusion complexes between CDs and the competitor (methyl orange, MO). The results are summarized in Table 1 in comparison with values from the literature.

**Table 1:** Formation constants (*K*_f_) of CD/MO and CD/PP inclusion complexes obtained by UV–visible spectroscopy using a spectral displacement method in comparison with values from the literature. Standard deviation values are <10%.

Formation constant (*K*_f_) M^−1^	log P^a^	α-CD	β-CD	HP-β-CD	RAMEB	CRYSMEB

MO	–	7810	2386	5597	15400	3594
*trans-*Anethole (**1**)	3.096	927, 1163^b^, 710^c^	542, 630^b^, 497^c^	845, 1042^b^ ,981^c^	1815, 1553^b^, 1110^c^	1039, 740^b^, 877^c^
Estragole (**2**)	2.818	335	987	1508	1916	1584
Isoeugenol (**3**)	2.379	178, 85^d^	364, 255^d^, 304^f^	418, 441^d^, 452^f^	514^d^, 547^f^	263^d^, 240^f,^
Eugenol (**4**)	2.100	350, 94^d^	462, 264^d^, 357^e^, 322^f^	436, 462^d^, 445^f^	568^d^ ,521^f,^	454^d^, 401^f,^
*p-*Coumaric acid (**5**)	1.43	1816	338	787	1030	668
Caffeic acid (**6**)	0.941	1540	318, 278^g^, 516^h^	526, 279^i^	991	404
Ferulic acid (**7**)	1.249	1769, 1162^j^	246, 205^k^	451, 590^k^	908	474

^a^from [22]; ^b^from [11]; ^c^from [23]; ^d^from [24]; ^e^from [25]; ^f^from [21]; ^g^from [26]; ^h^from [27]; ^i^from [28]; ^j^from [29]; ^k^from [30].

The stoichiometry of all studied inclusion complexes was found to be 1:1 (CD:PP). This is in accordance with results generally obtained for aromatic compounds. The calculated *K*_f_ values were in good agreement with those found in literature [11,21–30]. The higher *K*_f_ values obtained for **1** and hydroxicinnamic acids with α-CD could be attributed to their ability to adopt a planar conformation, allowed by the conjugation of the double bond with the aromatic ring. This conformation provides them an ideal geometry that easily penetrates and occupies the whole internal space of the smallest α-CD cavity. The β-CD derivatives present a better complexation capacity towards **2**, **3** and **4** than α-CD. Among β-CDs, RAMEB showed the highest formation constants. This result could be explained by the higher hydrophobic character of its cavity which favors inclusion complex formation [31].

It is well known that complexation with CDs depends on guest hydrophobicity (expressed by different descriptors) [32–35]. However, in our study, no clear correlation was found between the *K*_f_ values and the log P of the guest molecules, meaning that steric considerations have also to be taken into account. Indeed, for β-CD derivatives, phenylpropenes with an allyl group (**2** and **4**) present higher stability constants than the respective PPs with a propenyl group (**1** and **3** respectively). Considering the studied PPs, *p*-substituted compounds present higher stability constants than *o-,p*-substituted ones.

### Phase solubility studies

**Figure 2:**
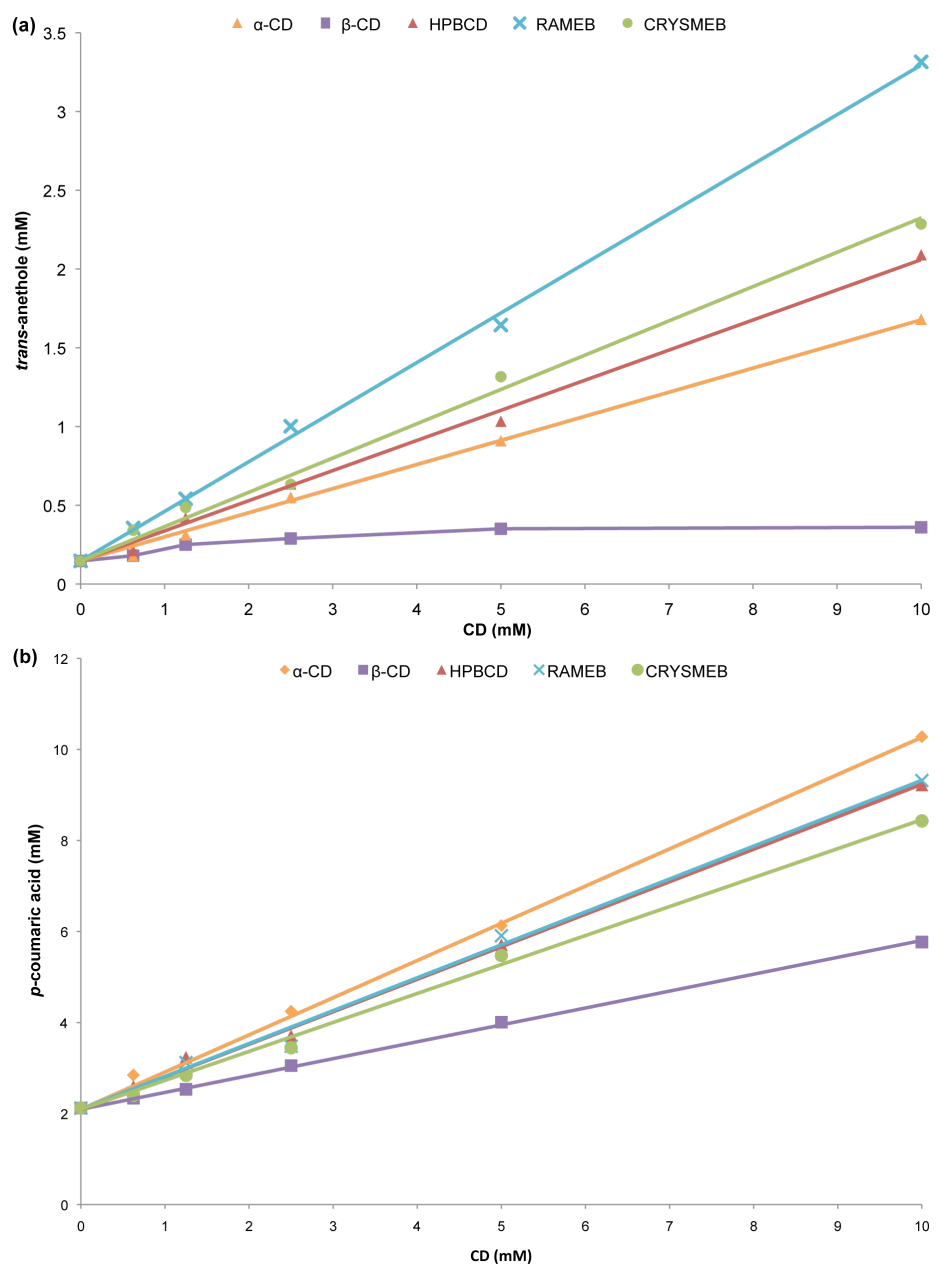
Phase solubility profiles of (a) CD/*trans*-anethole and (b) CD/*p*-coumaric acid inclusion complexes.

Figure 2 illustrates the phase solubility profile obtained for (a) CD/**1** and (b) CD/**5** inclusion complexes. A_L_-type profiles were obtained for all studied inclusion complexes except for β-CD/**1** and β-CD/**2** where B-type profiles were obtained (see Supporting Information File 1, Figure S1). Indeed, the B-profile is frequently observed for β-CD especially with poorly soluble drugs [19].

For all A_L_-type phase-solubility diagrams, the relative slope was less than unity confirming the formation of 1:1 inclusion complexes (in agreement with spectral displacement method). Thus, *K*_f_ values of the inclusion complexes (Table 2) were calculated from the slope and the intrinsic solubility (*S*_0_) of the PP in water according to Equation 1. For β-CD/**1** and β-CD/**2**, *K*_f_ values were determined using the linear portion of the corresponding phase solubility diagram. We can notice that *K*_f_ values obtained from the phase solubility profiles were generally in good agreement with those obtained by UV–visible except for **1**, the less soluble compound.

Indeed, according to Equation 1, the intrinsic solubility *S*_0_ should be equal to the intercept (*S*_int_) of the phase solubility diagram. However, for poorly soluble compounds (aqueous solubility <0.1 mM), the *S*_0_ is in general much larger than the *S*_int_ that leads to an overestimation of *K*_f_ values. Moreover, *K*_f_ values obtained from phase-solubility are generally apparent values because they combine several effects on the guest solubility: inclusion complexation, self-association of poorly soluble guests, self-aggregation of CD/guest complexes, as well as non-inclusion interaction and micelles formation [18].

Our findings were in good agreement with the literature and confirmed that the error in the determination of *K*_f_ values from the phase solubility diagrams increases with decreasing drug solubility, especially due to the inaccuracy of *S*_0_ determination for very poorly soluble drugs.

The presence of CDs considerably enhances the PPs solubility (up to 17 fold for **1** with a 10 mM RAMEB solution). Similar findings were found in literature [28,35–39]. The solubilizing effect of CDs can be evaluated using their complexation efficiency (CE), i.e., the concentration ratio between cyclodextrin in a complex and free cyclodextrin (Equation 2, Table 2). Importantly, CE is a more accurate parameter because it is independent of both *S*_0_ and *S*_int_ [18].

**Table 2:** Formation constants (*K*_f_), solubility enhancement ratio *S*_t_/*S*_0_, CE, optimum molar ratio and increase in formulation bulk of phenylpropanoids.

Guest	*S*_0_ (mg/L)	CD	*K*_f_ (M^−1^)	*S*_t_/*S*_0_^a^	CE	Molar ratio (PP:CD)	Increase in formulation bulk

*trans-*Anethole (**1**)	22	α-CD	1274	11	0.19	1:6.38	42
		β-CD	537	3	0.08	1:13.75	106
		HP-β-CD	1510	14	0.22	1:5.54	56
		RAMEB	2157	17	0.31	1:4.18	38
		CRYSMEB	1782	15	0.26	1:4.84	40

Estragole (**2**)	48	α-CD	682	4	0.22	1:5.54	36
		β-CD	882	3	0.28	1:4.51	35
		HP-β-CD	1412	11	0.46	1:3.19	32
		RAMEB	1694	12	0.55	1:2.83	26
		CRYSMEB	1512	10	0.49	1:3.05	25

Isoeugenol (**3**)	665	α-CD	110	2	0.45	1:3.24	19
		β-CD	210	1.5	0.85	1:2.18	15
		HP-β-CD	449	2.7	1.82	1:1.55	14
		RAMEB	428	2.6	1.73	1:1.58	13
		CRYSMEB	312	2.5	1.26	1:1.79	13

Eugenol (**4**)	1038	α-CD	246	2	1.56	1:1.64	10
		β-CD	513	2.1	3.25	1:1.31	9
		HP-β-CD	445	2.2	2.81	1:1.36	12
		RAMEB	550	2.2	3.48	1:1.29	11
		CRYSMEB	440	2.1	2.78	1:1.36	10

*p-*Coumaric acid (**5**)	344	α-CD	1988	4.8	4.17	1:1.24	7
		β-CD	306	2.8	0.64	1:2.56	18
		HP-β-CD	1099	4.4	2.30	1:1.43	13
		RAMEB	1228	4.4	2.57	1:1.39	11
		CRYSMEB	900	4	1.89	1:1.53	11

Caffeic acid (**6**)	300	α-CD	1819	5.5	3.03	1:1.33	7
		β-CD	425	3.5	0.71	1:2.41	15
		HP-β-CD	534	3.8	0.89	1:2.12	18
		RAMEB	825	4.4	1.37	1:1.73	13
		CRYSMEB	552	3.8	0.92	1:2.09	14

Ferulic acid (**7**)	333	α-CD	1737	5.2	2.98	1:1.34	7
		β-CD	326	3.1	0.56	1:2.79	16
		HP-β-CD	833	4.4	1.43	1:1.70	13
		RAMEB	1045	4.8	1.79	1:1.56	11
		CRYSMEB	512	3.8	0.88	1:2.14	13

^a^*S*_t_ and *S*_0_ are the guest solubility in 10 mM CD solution and in water, respectively.

The CE mean value for β-CD and its derivatives were 0.91, 1.42, 1.70 and 1.21 for β-CD, HP-β-CD, RAMEB and CRYSMEB, respectively. This confirms that β-CD derivatives are better solubilizers than native β-CD. Moreover, RAMEB seems to be the best solubilizer in agreement with literature [19,40].

CE values allowed the evaluation of the optimal PP:CD ratio in the complexation medium for the preparation of solid inclusion complexes as well as the increase in formulation bulk (Equation 3 and Equation 4, Table 2). These are crucial parameters that reveal the CD concentration needed to solubilize the guest as well as the possibility of using CDs in the formulation of solid dosage forms [18].

**Figure 3:**
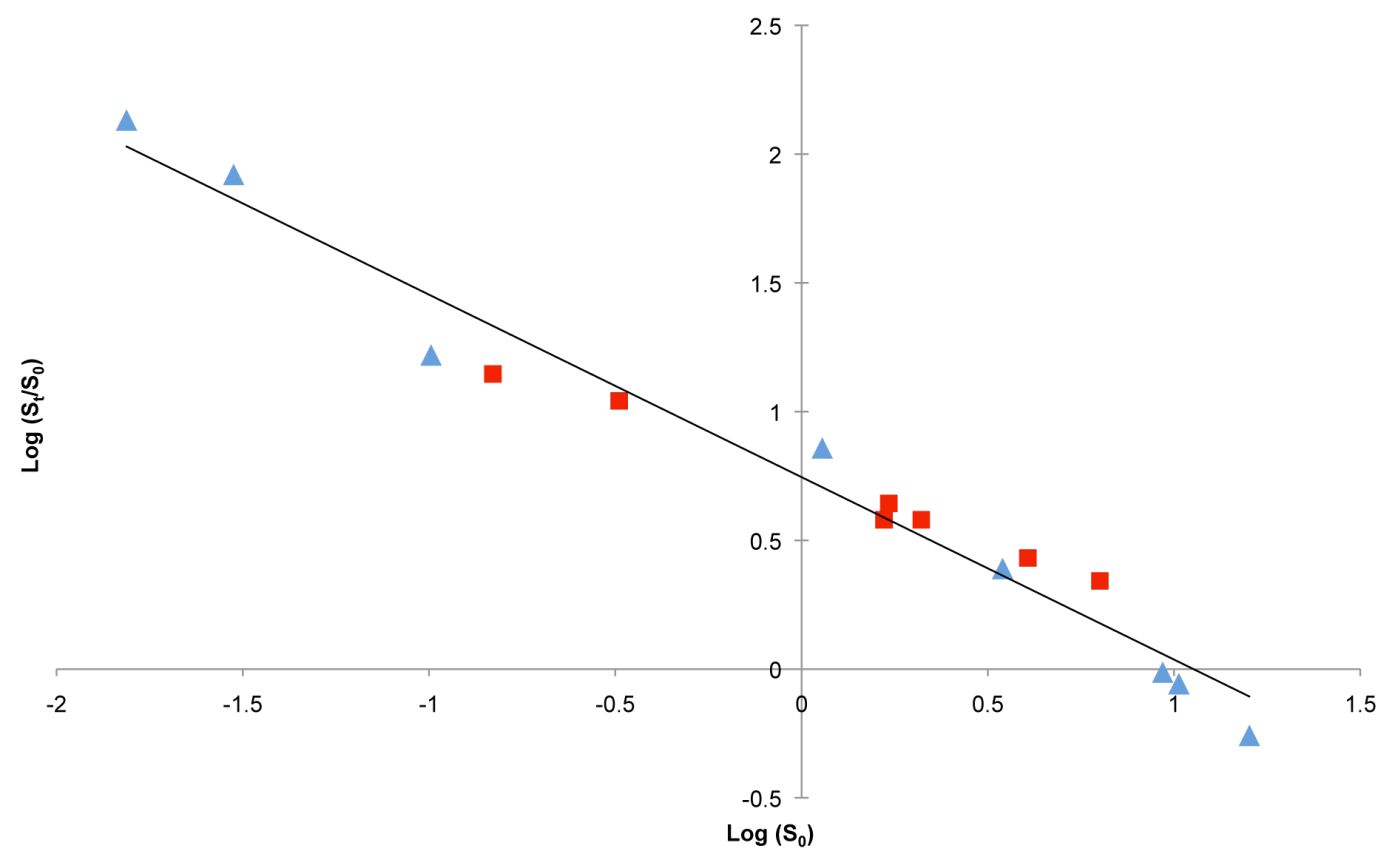
Solubility enhancement (log (*S*_t_/*S*_0_)) as a function of the solubility (log (*S*_0_)) of studied phenylpropanoids (red squares) and monoterpenoids (blue triangles) [32].

Finally, the solubility enhancement (*S*_t_/*S*_0_) in the presence of CD was evaluated. Literature lacks noticeable information on the influence of the guest *S*_0_ on the solubilizing effect of CD. In order to obtain a relevant guest range, we also introduced our previous results obtained for 8 monoterpenes in the presence of HP-β-CD [32]. Figure 3 illustrates the plot of log (*S*_t_/*S*_0_) in the presence of 10 mM of HP-β-CD as a function of guest log (*S*_0_). A linear relationship was found proving that the formation of inclusion complexes occurs through desolvation/dissolution of guest molecules. Furthermore, this observation confirms the fact that the solubilizing effect of CD greatly increases with the decrease in guest solubility. Deviation from this correlation could be allocated to the formation and precipitation of inclusion complexes with limited aqueous solubility especially with less soluble natural CDs.

### Molecular modeling

A molecular modeling study was performed to find out the most probable conformation of the β-CD/PP complexes and to give a meaningful 3D visualization of the complexes. Two docking strategies were used: i) inclusion into the CD cavity through the propenyl or allyl side chain of PP and ii) inclusion through the hydroxy or methoxy group. In both cases guests are allowed to penetrate the cavity through the wider rim of the CD. The computed complexation energies (Δ*E*) were calculated upon the docking of each PP into the β-CD cavity. The energy variation (Δ*E*) was used to find the most stable configurations of the inclusion complexes.

**Figure 4:**
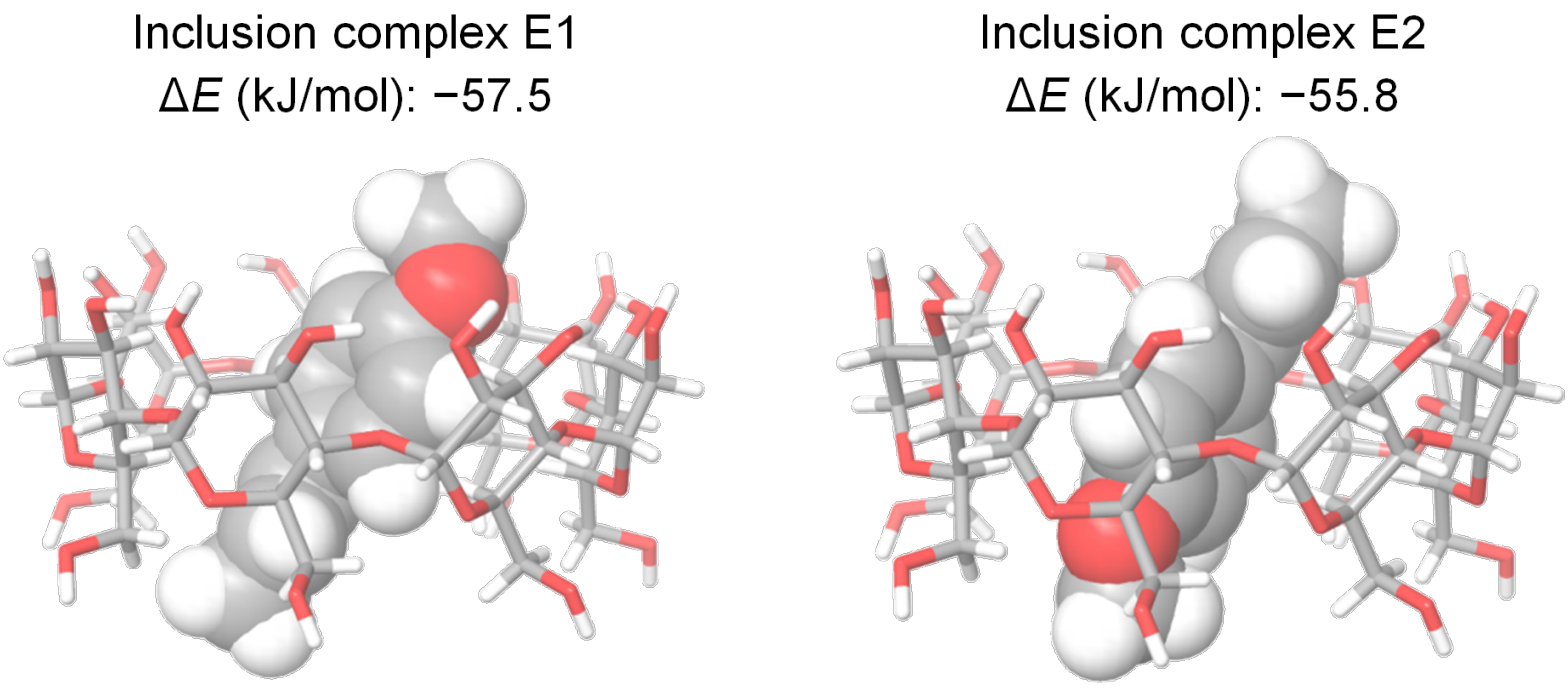
Representation of the most stable CD/*trans*-anethole inclusion complex conformers resulting from the two docking strategies.

The most stable conformations obtained from the two docking strategies and the corresponding energies (Δ*E*) are shown in Figure 4 in the case of **1** (see Supporting Information File 1, Table S1 for other compounds).

No preferential inclusion mode was observed, the result being in agreement with previous NMR results [23]. Molecular modeling demonstrated that the aromatic ring and the side chain of PPs were embedded inside the cavity of β-CD, leaving the more polar groups exposed at the periphery of the cavity. These results confirm that formation of inclusion complexes between CD and aromatic guests occurs by the prevalence of hydrophobic interactions and are in good agreement with literature data [21,28–30,41].

### Determination of DPPH radical scavenging activity

**Figure 5:**
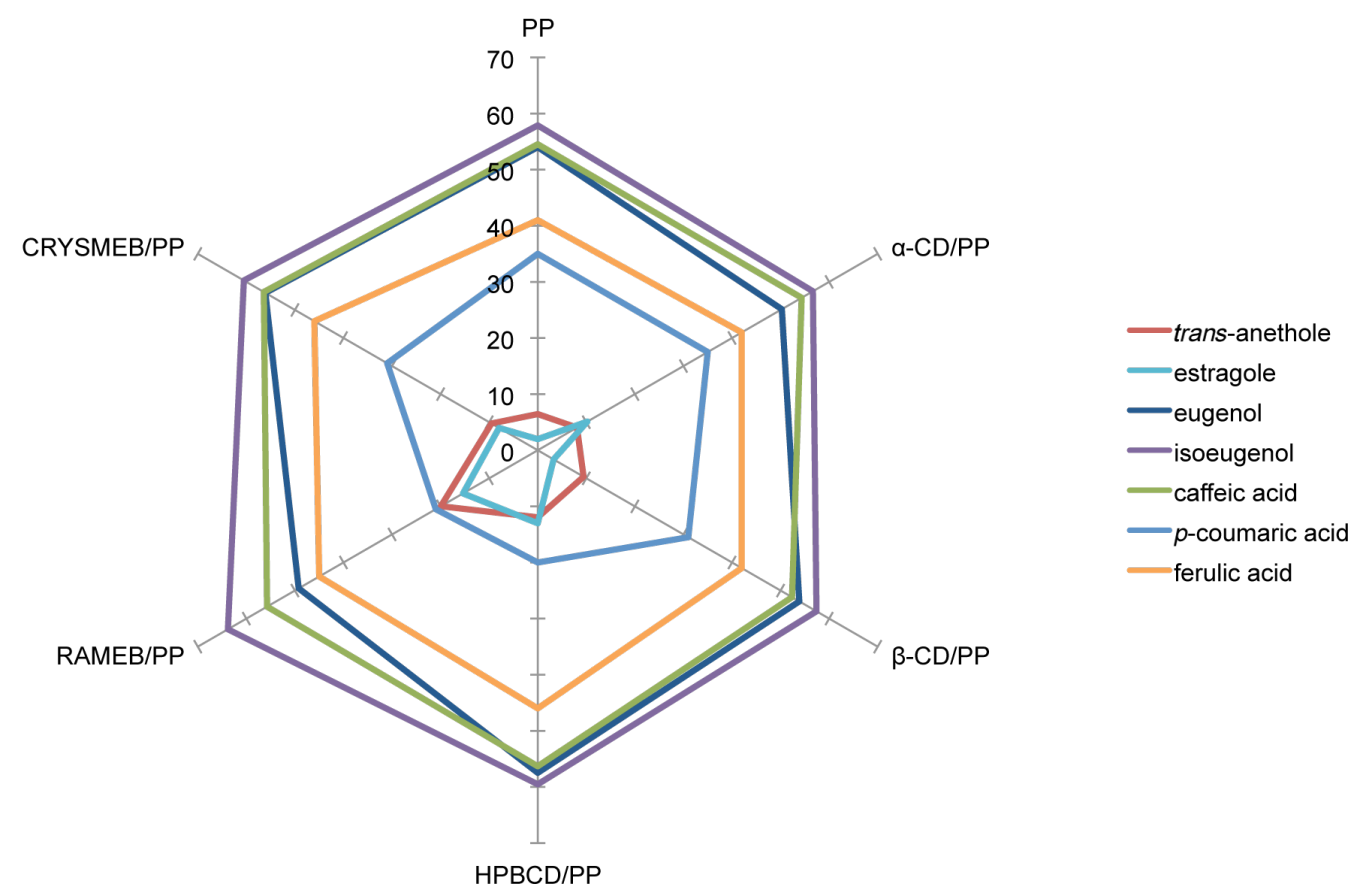
DPPH radical scavenging activity (%) of studied PPs alone or in presence of CD.

The DPPH^•^ assay was used to measure the radical scavenging activity of PPs. Depending on the studied PP, two types of kinetic behavior were observed. Isoeugenol (**3**), eugenol (**4**), caffeic acid (**6**) and ferulic acid (**7**) reacted rapidly with DPPH^•^ while *trans-*anethole (**1**), estragole (**2**) and *p-*coumaric acid (**5**) had a slow kinetic behavior. These observations were consistent with the literature data [42]. Results obtained in the absence and presence of CDs, are presented in Figure 5.

Isoeugenol (**3**), eugenol (**4**), *p-*coumaric acid (**5**), caffeic acid (**6**) and ferulic acid (**7**) presented higher antioxidant activity than *trans-*anethole (**1**) and estragole (**2**). It is well known that the antioxidant activity of phenolic compounds such as PPs depends mainly on the degree of hydroxylation. This could explain why **1** and **2**, which carry no hydroxy group, reacted little with DPPH^•^. The activity of **3**, **4**, **5**, **6** and **7** was kept unchanged upon complexation with CDs. This indicated that CDs did not interfere with the active groups of these PPs during inclusion complex formation which was still in turn available to react with DPPH radicals. An increase in activity of **1** and **2** in the presence of CDs was observed. It could be explained by the formation of intermolecular hydrogen bonds with the hydroxyl groups of CD, resulting in the formation of a stable phenoxyl radical [43–44].

## Conclusion

In this work, experimental and theoretical studies for CD inclusion complexes with seven PPs were performed. Our results clearly demonstrated that all CDs could form 1:1 stable inclusion complexes with studied PPs. We showed that the stability of the inclusion complexes depends on the cavity size of CD and on the molecular structure and shape of the guest. A linear relationship was found between the solubility of the guest (*S*_0_) and the improvement of the solubility upon complexation with CD (*S*_t_/*S*_0_). DPPH^•^ scavenging assays showed that the antioxidant activity of PPs was retained upon complexation.

## Experimental

### Materials

*trans-*Anethole (99%) and 2,2-diphenyl-1-picrylhydrazyl were purchased from Aldrich. Estragole (analytical standard) was provided by Fluka Chemicals. Isoeugenol (99%), eugenol (99%), *p*-coumaric acid (≥98%), caffeic acid (≥98%), ferulic acid (99%) and methyl orange (MO) were purchased from Acros Organics. α-CD, β-CD, HP-β-CD (DS = 5.6) and RAMEB (DS = 12.6) were purchased from Wacker-Chemie (Lyon, France). CRYSMEB (DS = 4.9) was provided from Roquette Frères (Lestrem, France). Distilled deionized water was used throughout this work.

### Formation constants (*K*_f_)

The UV–visible competition method with methyl orange (MO) as competitor was carried out according to the method described by Landy et al. [20]. In order to calculate CD/PP *K*_f_ values, CD/MO inclusion complexes were firstly characterized by a direct titration method. Then, a spectral displacement method was applied by adding PP to a solution containing constant concentration of MO and CD. The addition of PP implied an absorbance increment leading to the calculation of CD/PP *K*_f_ values. Spectra were recorded between 520–530 nm for a MO concentration fixed at 0.1 mM. This wavelength range corresponds to the optimal spectral variation between the absorbance of free and complexed forms of MO. All *K*_f_ values were calculated by an algorithmic treatment applied to the first derivatives of UV spectra in order to avoid any spectral influence of diffraction phenomena. Absorption studies were performed using a UV–visible dual-beam spectrophotometer (Perkin Elmer Lambda 2S) with a 1 cm thick quartz cuvette.

### Phase solubility studies

Phase solubility studies were carried out according to the method described by Higuchi and Connors [45]. Excess amount of PP was added to 1 mL of CD solution at different concentrations ranging from 0 to 10 mM. The mixtures were shaken at 25 °C for 24 h then filtered through a 0.45 μm membrane filter. The concentration of PP in the filtrate was determined spectrophotometrically at the maximum wavelength corresponding to each PP. Phase solubility diagrams were obtained by plotting the solubility of PP as a function of CD concentration. *K*_f_ value of CD/PP inclusion complex was calculated from the linear segment of the phase–solubility diagrams using the following equation:

[1]
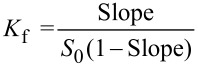


where *S*_0_ is the intrinsic solubility of PP in the absence of CD and the Slope is the slope of the phase–solubility profile. The solubilizing potential of CD was evaluated by the complexation efficiency (CE) parameter. CE equal the complex to free CD concentration ratio and was calculated from the slope of the phase solubility diagram [18]:

[2]
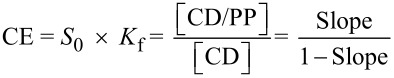


where [CD/PP] is the concentration of dissolved inclusion complex and [CD] is the concentration of dissolved free CD. The CE can be used to calculate the PP:CD ratio as follows:

[3]
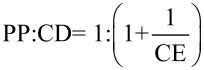


The correlation between the CE and the molecular weight of CD or guest allowed the determination of the increase in formulation bulk which can be calculated using the following equation:

[4]
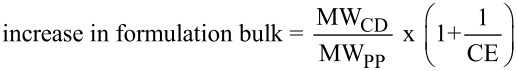


where MW_CD_ and MW_PP_ are the molecular weight values of CD and PP, respectively.

### Molecular modeling

Molecular modeling of inclusion complexes was realized by means of Macromodel with MMFFs force field in the presence of water (GB/SA implicit model). The host structure was based on a nondistorted symmetrical β-CD. PP structures were constructed manually and minimized, prior to inclusion simulations. The docking of each PP inside CD was realized by means of conformational Monte Carlo searches, with the generation of 5000 conformations (FMNR conjugate gradient minimization, convergence fixed to 0.01 kJ Å^−1^ mol^−1^). During the search, CD was maintained rigid, while the PP was freely modified. For each PP, two types of inclusion complexes according to the two docking strategies were explored (E1 and E2 which refers to the penetration of the CD cavity by the PP via the propenyl or allyl chain moiety or other phenyl ring substituents, respectively) leading to two different orientations of the aromatic ring inside the host cavity. The total energy difference (Δ*E*, kJ/mol) between inclusion complexes and the sum of their individual components in their optimized fundamental states was calculated (Δ*E* = *E*_CD/PP_ – (*E*_CD_ + *E*_PP_)) and used as the theoretical parameter to evaluate the complexation energy of the inclusion complex.

### DPPH radical scavenging method

The 2,2-diphenyl-1-picrylhydrazyl (DPPH) radical scavenging activity of PP and their inclusion complexes was measured using the method described by Brand-Williams, Cuvelier, and Berset (1995) [42] with some modifications. 1 mg of PP was dissolved in 50 mL of water, or 10 mM CD solution. 2 mL aliquot of the solution was vigorously mixed with 2 mL of ethanolic DPPH^•^ solution (0.1 mM). Depending on the kinetic behavior of PP, mixtures were shaken for 1 h (isoeugenol (**3**), eugenol (**4**), caffeic acid (**6**) and ferulic acid (**7**)) or 15 h (*trans-*anethole (**1**), estragole (**2**) and *p-*coumaric acid (**5**)) and allowed to stand at 25 °C in the dark. The absorbance was measured at 520 nm. Blank sample was prepared by mixing 2 mL of distilled water with 2 mL of DPPH^•^ solution. The scavenging activity was calculated using the following equation:

[5]
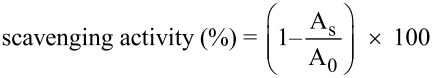


where *A*_s_ and *A*_0_ are the absorbance of sample and blank sample, respectively.

## Supporting Information

File 1 Phase solubility profiles of CD inclusion complexes and most stable CD/PP inclusion complex conformers.
