# Supplementary material for: Effect of cyclodextrin complexation on phenylpropanoids’ solubility and antioxidant activity
Source: Beilstein J Org Chem. 2014 Oct 6;10:2322–31. doi: 10.3762/bjoc.10.241 (PMC4187030; doi:10.3762/bjoc.10.241)
Supplement: File 1 — Phase solubility profiles of CD inclusion complexes and most stable CD/PP inclusion complex conformers. [file Beilstein_J_Org_Chem-10-2322-s001.pdf]

Supporting Information

for

Effect of cyclodextrin complexation on phenylpropanoids' solubility and antioxydant activity

Miriana Kfoury[1,2,3], David Landy[2,3], Lizette Auezova[1], Hélène Greige-Gerges[1]

and Sophie Fourmentin[2,3] *

Address: [1]Bioactive Molecules Research Group, Doctoral School of Science and Technology, Department of Chemistry and Biochemistry, Faculty of Sciences-2, Lebanese University, Lebanon, [2]Univ Lille Nord de France, F-59000 Lille, France and [3]ULCO, UCEIV, F-59140 Dunkerque, France

Email : Sophie Fourmentin - lamotte@univ-littoral.fr

* Corresponding author

Phase solubility profiles of CD inclusion complexes and most stable CD/PP inclusion complex conformers

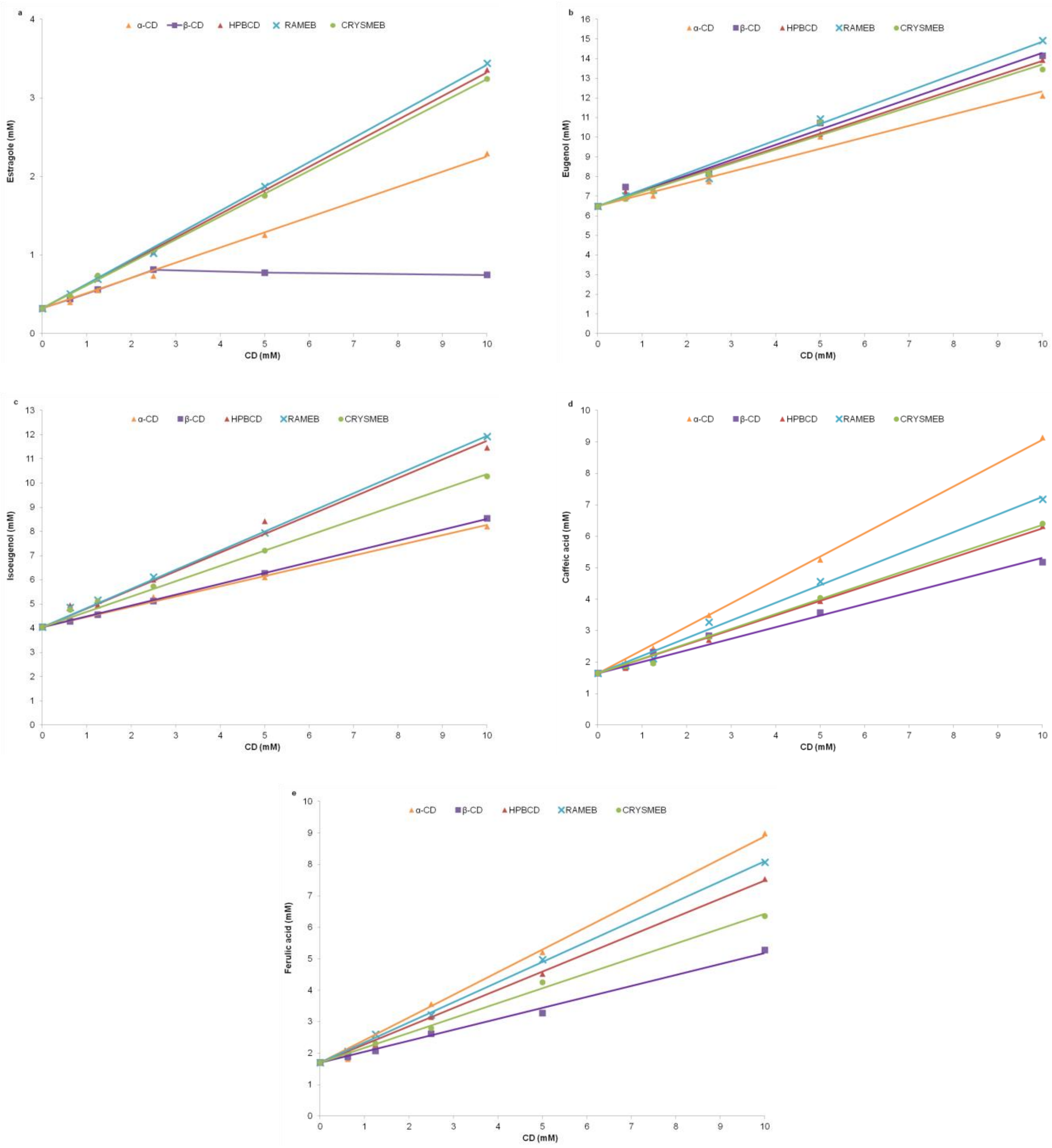

Figure S1: Phase solubility profiles of a) CD/estragole, b) CD/eugenol, c) CD/isoeugenol, d) CD/caffeic acid and e) CD/ferulic acid inclusion complexes.

Table S1: Representation of the most stable CD/PP inclusion complex conformers resulting from the two docking strategies.

Host/guest	Δ*E* (kJ/mol)	
	Inclusion complex E1	Inclusion complex E2
β-CD/ estragole	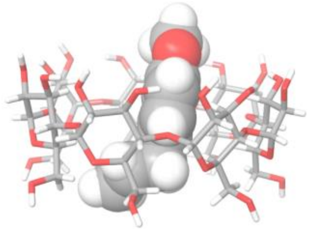 -46.0	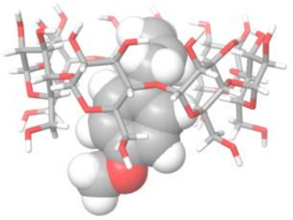 -43.6
β-CD/ eugenol	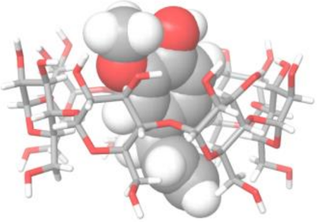 -45.2	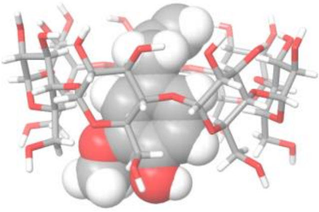 -46.7
β-CD/ isoeugenol	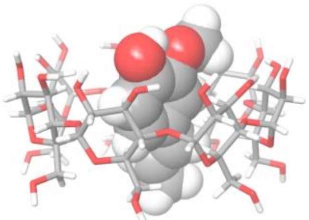 -50.9	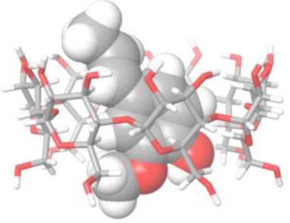 -49.9
β-CD/ caffeic acid	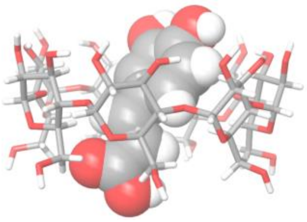 -33.3	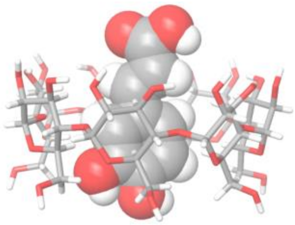 -34.6
β-CD/*p*-coumaric acid	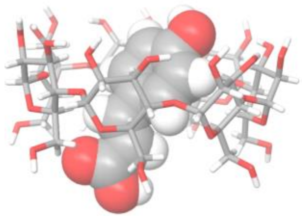 -38.4	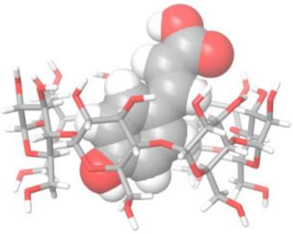 -38.8
β-CD/ ferulic acid	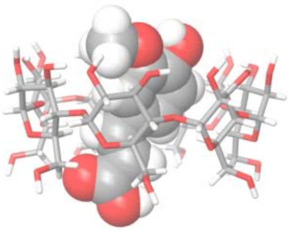 -32.2	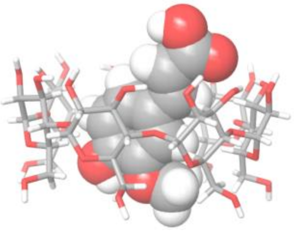 -28.1
